# Pre-surgery planning tool for estimation of resection volume to improve nasal breathing based on lattice Boltzmann fluid flow simulations

**DOI:** 10.1007/s11548-021-02342-z

**Published:** 2021-03-24

**Authors:** M. Berger, M. Pillei, A. Giotakis, A. Mehrle, W. Recheis, F. Kral, M. Kraxner, H. Riechelmann, W. Freysinger

**Affiliations:** 1 Department of Environmental, Process and Energy Engineering, MCI-The Entrepreneurial School, Innsbruck, Austria; 2 Department of Otorhinolaryngology-Head and Neck Surgery, Medical University of Innsbruck, Innsbruck, Austria; 3 Department of Fluid Mechanics, Friedrich-Alexander-University Erlangen-Nuremberg, Erlangen, Germany; 4 Department of Mechatronics, MCI-The Entrepreneurial School, Innsbruck, Austria; 5 University Hospital of Radiology, Medical University Innsbruck, Innsbruck, Austria

**Keywords:** Breathing improvement, Nasal cavity, Optimization, Postoperative outcome

## Abstract

**Purpose:**

State-of-the-art medical examination techniques (e.g., rhinomanometry and endoscopy) do not always lead to satisfactory postoperative outcome. A fully automatized optimization tool based on patient computer tomography (CT) data to calculate local pressure gradient regions to reshape pathological nasal cavity geometry is proposed.

**Methods:**

Five anonymous pre- and postoperative CT datasets with nasal septum deviations were used to simulate the airflow through the nasal cavity with lattice Boltzmann (LB) simulations. Pressure gradient regions were detected by a streamline analysis. After shape optimization, the volumetric difference between the two shapes of the nasal cavity yields the estimated resection volume.

**Results:**

At LB rhinomanometry boundary conditions (bilateral flow rate of 600 ml/s), the preliminary study shows a critical pressure gradient of −1.1 Pa/mm as optimization criterion. The maximum coronal airflow Δ*A*  := cross-section ratio $\frac{\mathrm{virtual surgery }}{\mathrm{post}-\mathrm{surgery}}$ found close to the nostrils is 1.15. For the patients a pressure drop ratio ΔΠ  := (pre-surgery − virtual surgery)/(pre-surgery − post-surgery) between nostril and nasopharynx of 1.25, 1.72, −1.85, 0.79 and 1.02 is calculated.

**Conclusions:**

LB fluid mechanics optimization of the nasal cavity can yield results similar to surgery for air-flow cross section and pressure drop between nostril and nasopharynx. The optimization is numerically stable in all five cases of the presented study. A limitation of this study is that anatomical constraints (e.g. mucosa) have not been considered.

## Introduction

Structural deformities within the human nasal cavity (e.g. septal deviation) frequently cause nasal obstruction. Functional nasal surgery is planned, based on the surgeon’s experience, using state-of-the-art investigation technique including 4-phase rhinomanometry [1]. Rhinomanometry allows separate measurements of left and right nasal cavities of the pressure drop between nostril and nasopharynx at various flow rates, however without information about the site of obstruction [2]. Septal surgery has a widely varying subjective patient success rate between 45% and 85% [3, 4].

Computational fluid dynamic simulations (CFD) based on the finite volume method (FVM) and lattice Boltzmann (LB) are nowadays performed on graphical processing units (GPU) in reasonable time [5, 6]. CFD of the nasal airflow is often based on FVM with complex meshing [7]. In comparison, CT data structure allows immediate use in LB simulation. Alternatively, LB features stable computation [8] for small Reynolds numbers [9]. Both simulation approaches were recently validated experimentally in [10, 11].

LB features advantages for optimizing respiratory flow with Cartesian meshes and isotropic grid spacing in Sailfish CFD [6] and grid cells can simply be added or removed from the fluid domain. LB is preferred over FVM, since it reached good agreement to experimental data for the nasal cavity [12].

Optimization in CFD is well developed [13], but has not been used on nasal breathing phenomena, to the best of our knowledge. However, the correction of the nasal septum to improve nasal breathing was performed with MATLAB [14]. DigBody^®^ [15] is a virtual surgery environment of MeComLand^®^ [16], a CFD tool to simulate nasal airflow without optimization feature that can plan virtual surgery through direct modification of the nasal passageway.

Compared to [14] the presented optimization process is not limited to the septum and compared to [15] LB simulations are used to determine pressure gradient regions based on automatically segmented CT data.

A code to optimize the shape of the nasal cavity based on fluid flow analysis is presented. LDA validated LB simulations [12] are used to find high pressure gradient regions (HPGRs).

## Methods

Five pre- and postoperative anonymous CT data sets (Siemens Somatom, beam current 88 mA, convolution kernel H30s, spatial resolution 0.38 × 0.38 × 0.6 mm^3^) were used. The study was conducted in accordance with local ethical guidelines as stipulated by the seventh revision of the Declaration of Helsinki. Anonymous CT data with the indication of septoplasty surgery were used. Postoperative CT data originated from other clinical exams or recurrences. No ethics committee approval for this anonymized retrospective study was needed.

**Fig. 1:**
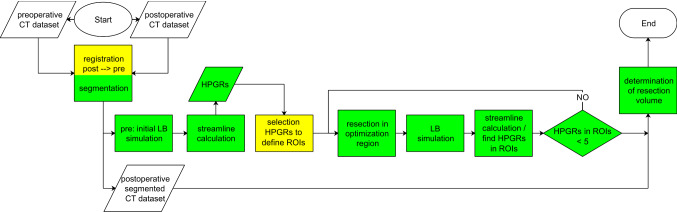
Block diagram of the optimization tool to determine the resection volume with LB simulations. *Green*: automatized, *yellow*: manual. HPGR, ROI, see text

Figure 1 shows the steps of the python-based optimization tool. Automatized blocks (green), yellow blocks require user interaction.

### CT segmentation

The output of segmentation defines the fluid domain of the simulation. The resolution for the LB simulations, 0.234 mm, was found by a mesh convergence study [12]. CT data were isotropically resampled (python ndimage.interpolation.zoom [17]) to 0.234 mm, thresholding at −300 Hounsfield [18, 19] units for airway segmentation, and binarized. Inlet and outlet boundary conditions for nostrils and nasopharynx were set as a sphere with a diameter of 70 mm overlapping both nostrils and a cuboid (60 × 40 × 30 mm^3^), respectively. The sphere’s center was defined in coronal slices just anterior to the tip of the nose. Region growing with a seed point inside the nasal airway passage was used to segment air. In the most inferior axial slice, the centroid of the air voxels defined the midpoint of the cuboid. The surface voxels of sphere and cuboid were saved as label maps as boundary conditions for LB simulations.

### Initial LB simulation

At the cuboid a Dirichlet velocity boundary condition (Sailfish CFD: NTRegularizedVelocity [6]) of bilateral inhalation flow rate of 600 ml/s was set, the pressure boundary condition on the sphere was set to ambient pressure (Sailfish CFD: NTDoNothing [6]), on solid surfaces the NTWallTMS [6] boundary condition was used. All voxels were initialized with $\vec{v} = \vec{0}$ m/s, simulation stopped when the airflow was fully developed, i.e. the pressure drop between inlets and outlet, after 0.0125 s [12]. The flow is characterized by a Reynolds number (*Re*) [9] of about 2800 based on the hydraulic diameters at the nostrils and the volume flux. The large eddy simulation (LES) turbulence model Smagorinsky [6] (with constant cs = 0.14, found in [12]) was used to simulate the transitional and unsteady features of nasal airflow [20] with the D3Q19 lattice element [6]. Computational time on a NVIDIA^®^ RTX 2080 TI was < 5 min. All LB simulations were performed with 13,605 time steps and a mesh with about 9 million cells.

### Streamline calculation

Velocity streamlines were calculated with Paraview’s *stream tracer tool* [21]. Continuous streamlines from inlet to outlet were generated, integration direction was set to *both*, integrating with Runge–Kutta 4–5. The streamline analysis is based on the last simulation result of the unsteady simulation with a maximum streamline length of 0.3 m, exceeding the size of the nasal cavity [22]. With Paraview option “Seed Type: Point Source” [21] 532 streamline start points, found in a preliminary study (Fig. 4), were randomly positioned within the sphere at the nostrils.

### Suggestion and selection of HPGRs

A high pressure gradient region indicates a constriction [9], a region with increased nasal resistance. A differential pressure criterion $\frac{{{\text{d}}p}}{{{\text{d}}l}}_{{{\text{streamline}}\;{\text{healthy}}}}$ (*p* … pressure, *l* … streamline) along the streamlines was determined in a preliminary study of five healthy persons without nasal septum deviation to define a baseline, Fig. 3. When the differential pressure at a streamline locally exceeds $\frac{{{\text{d}}p}}{{{\text{d}}l}}_{{{\text{streamline}}\;{\text{healthy}}}}$ a HPGR is identified at this position.

**Fig. 2:**
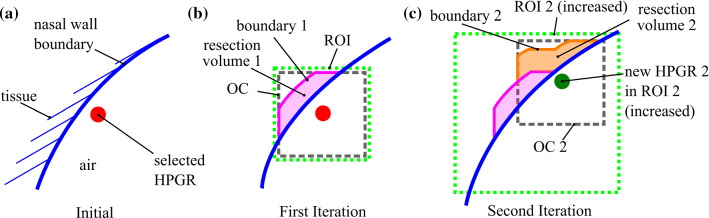
Schematic of optimization. **a** Beginning: one selected HPGR. *Blue line* is the nasal wall boundary. **b** First iteration: the wall boundary condition inside the optimization cube (OC) is moved in surface normal direction to the OC wall, creating resection volume 1. Here ROI and OC have the same size, HPGR is placed in the center of OC. Every iteration increases ROI in all six cube directions by 0.234 mm. **c** Second Iteration: HPGR 2 was found inside ROI 2. HPGR 2 was positioned at the center of OC creating boundary 2 and resection volume 2. LB simulation was performed with added resection volumes 1 and 2. The resection volume is determined by subtracting initial and final optimized nasal airflow cross sections

**Fig. 3:**
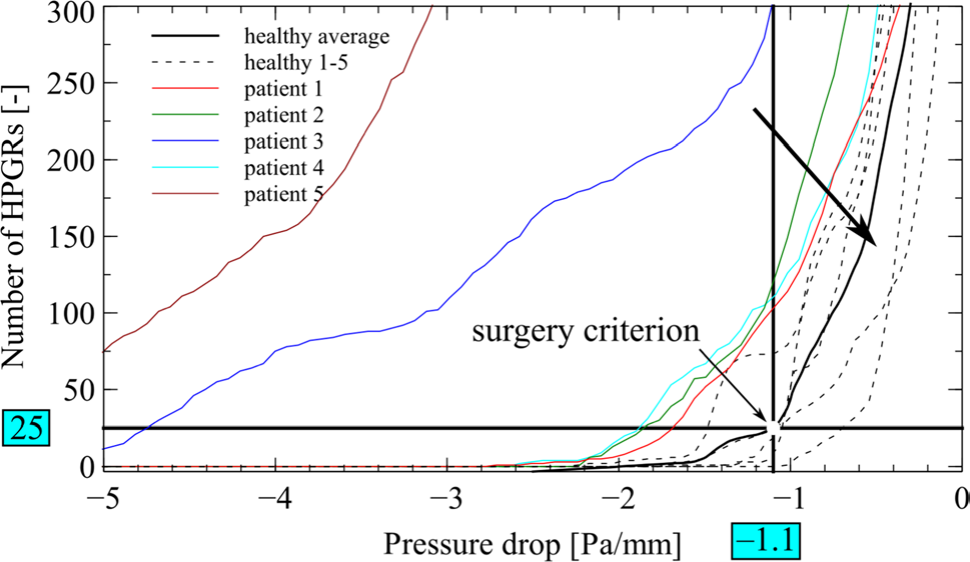
Results of the preliminary study to find the surgery criterion. Healthy persons had consistently less HPGRs than patients. Patients 3 and 5 had a pronounced septum deviation, HPGRs starting < −5 Pa/mm [12]. HPGRs are evaluated along the generated streamlines. Arrow indicates the domain “healthy”.

The pressure gradient was iteratively changed over the extracted streamlines [−5, 0] Pa/mm in steps of −0.1 Pa/mm to find the critical locations. Figure 3 shows local pressure variations. HPGRs based on initial fluid flow simulation are used for optimization. Desired surgery points were selected by the user in the set of HPGRs to define the regions of interests (ROI) (see Fig. 2) with a graphical user interface, Fig. 10.

### Optimization

Every HPGR is placed in a ROI and an optimization cube (OC) of 10 mm side length, Fig. 2b. During optimization, the wall (see Fig. 2a,b—blue line) is moved inside the ROI in surface normal direction towards the boundary. Laplace filtering (scipy.ndimage.filters.laplace [17]) detects edges of the dataset; adding this edge the “wall” is moved by one voxel, yielding resection volume 1 (see Fig. 2b).

Initially ROI coincides with OC. In every iteration ROI size increases by 0.468 mm. HPGRs are recalculated at every optimization step and considered for optimization inside the ROIs (see Fig. 2c). Every iteration is initialized with the initial simulation result, new volumes with $\vec{v} = \vec{0}$ m/s; LB simulation stopped when pressure drop between inlets and outlet became fully developed after 0.00625 s. Optimization stops when < 5 HPGRs are found in all ROIs. Figure 2c shows the resulting resection surface after two iterations.

### Mesh independence

The solutions of the simulations are required to be mesh independent [23]. In all simulations the surface averaged pressure $\frac{{\smallint p {\text{d}}A}}{A}$ at the investigation planes (see Figs. 5, 6) for every patient was used. The grid convergence index (GCI) [23] was used to determine the adequate lattice resolution and was used in this study.

## Results

### Results of the mesh independence study

Table 1 shows the results of the mesh independence study (for details please see [12]) for the five patients with pre- and post-surgery LB simulations with 0.0125 s integration time. All simulations showed asymptotic grid convergence with *p* > 1 and for all simulations ${\text{MI}}:\, = \,\frac{{gci_{23} }}{{r^{p} gci_{12} }} \approx 1,$ mesh independence is valid [23].

**Table 1:** Results of mesh independence study for the five patient pre-surgery LB simulations

	*p*	${f}_{0}\, [\mathrm{Pa}]$	MI
Patient 1 pre	5.89	−75.54	0.994
Patient 1 post	4.38	−45.79	0.987
Patient 2 pre	3.57	−22.78	0.956
Patient 2 post	1.05	−14.13	0.997
Patient 3 pre	2.01	−53.25	0.974
Patient 3 post	5.18	−29.13	1.015
Patient 4 pre	13.89	−12.34	1.048
Patient 4 post	1.52	−6.64	0.971
Patient 5 pre	35.12	−112.49	1.012
Patient 5 post	19.06	−59.14	1.014

*p* order of convergence; ${f}_{0}$ extrapolated pressure at grid resolution of 0 mm, *MI* mesh convergence index

### Streamline analysis to establish a surgery criterion

HPGRs were identified by a preliminary study on anonymous CT datasets of five patients with and five without septum deformation (Fig. 3). The onset is the first appearance of HPGRs, at ≂2.5 Pa/mm (patients 1, 2 and 4) and < ≂5 Pa/mm for the rest. Healthy persons show an onset of HPGRs at smaller values, see Fig. 3. The HPGRs for healthy individual were averaged, thick black curve. 25 HPGRs at −1.1 Pa/mm were chosen as a surgery criterion (Fig. 3) as this created a regime (arrow in Fig. 3) that visually separated patients from healthy individuals well.

**Fig. 4:**
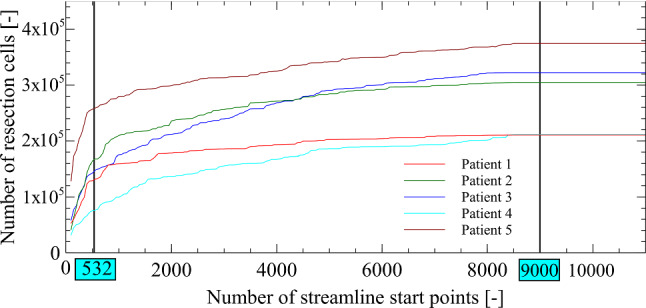
Preliminary convergence study to find the minimum number of streamlines to cover resection cells

In a second preliminary study, the number of streamlines was determined to cover a sufficient amount of the resection volume per optimization iteration. Streamlines number was varied from 0 to 10,000 in steps of 50 and was randomly placed at the inlet sphere. 9000 streamline starting points gave a stationary solution (Fig. 4) within 5 min per patient at 532 streamline start points, at least 50% of the total amount of resection volume is covered within 20 s. As a trade-off between speed and accuracy 532 streamline starting points were chosen for the simulations. This is further justified as the first steep increase of detected resection cells is covered. A larger number of starting points were not deemed feasible for this initial investigation.

### LB simulation results, detected HPGRs

**Fig. 5:**
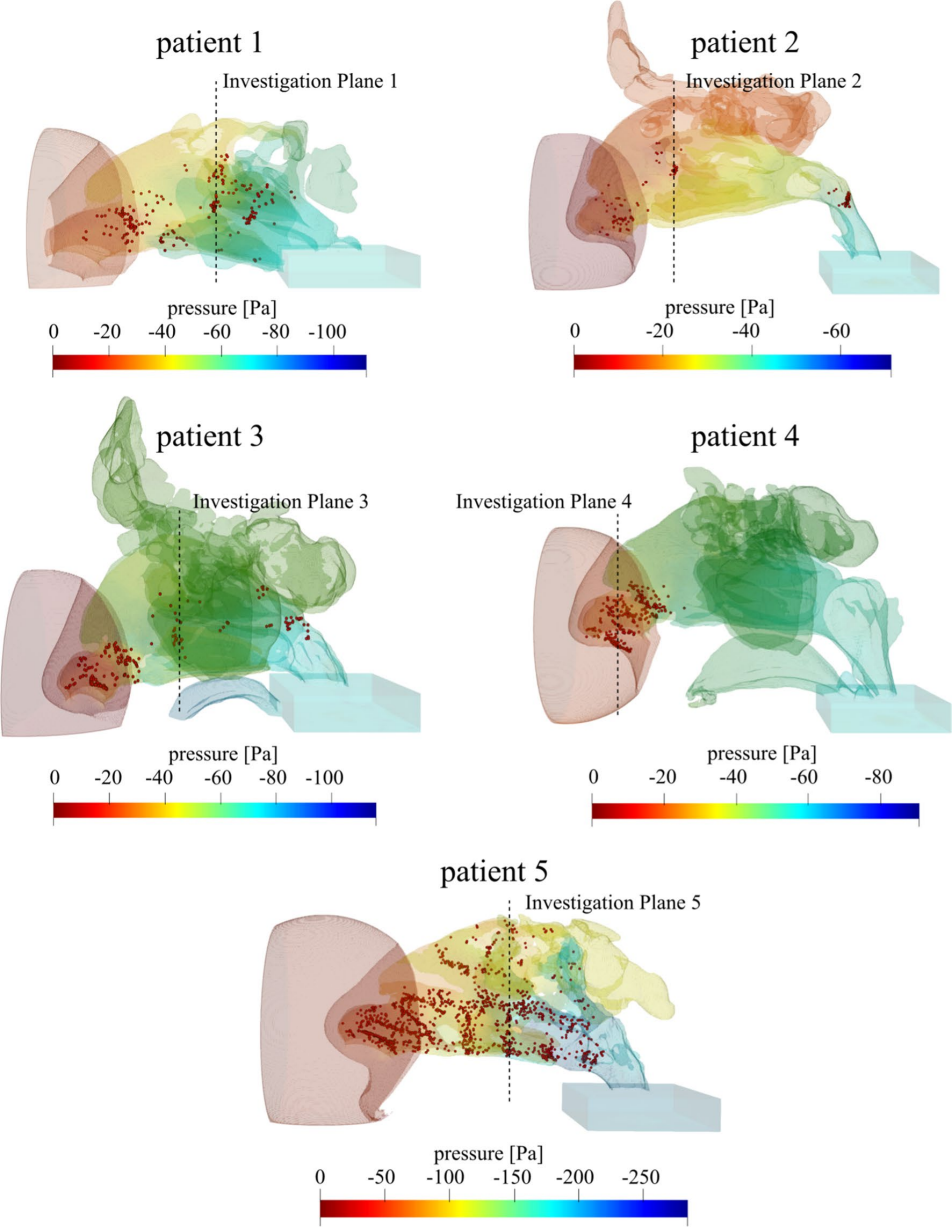
Simulation results of patients with nasal septum deviation of pre-surgery CT datasets. *Colors* depict static pressure in Pa. Investigation planes were defined on coronal planes with high concentration of HPGRs, *red* spheres

LB simulation results on preoperative CT data of the five patients with nasal septum deviation are shown in Fig. 5. The color gradient depicts the static pressure drop originating from anatomical constrictions. Investigation planes were chosen at coronal positions with high concentration of HPGRs (Fig. 5). These positions were varied to test the proposed optimization.

**Fig. 6:**
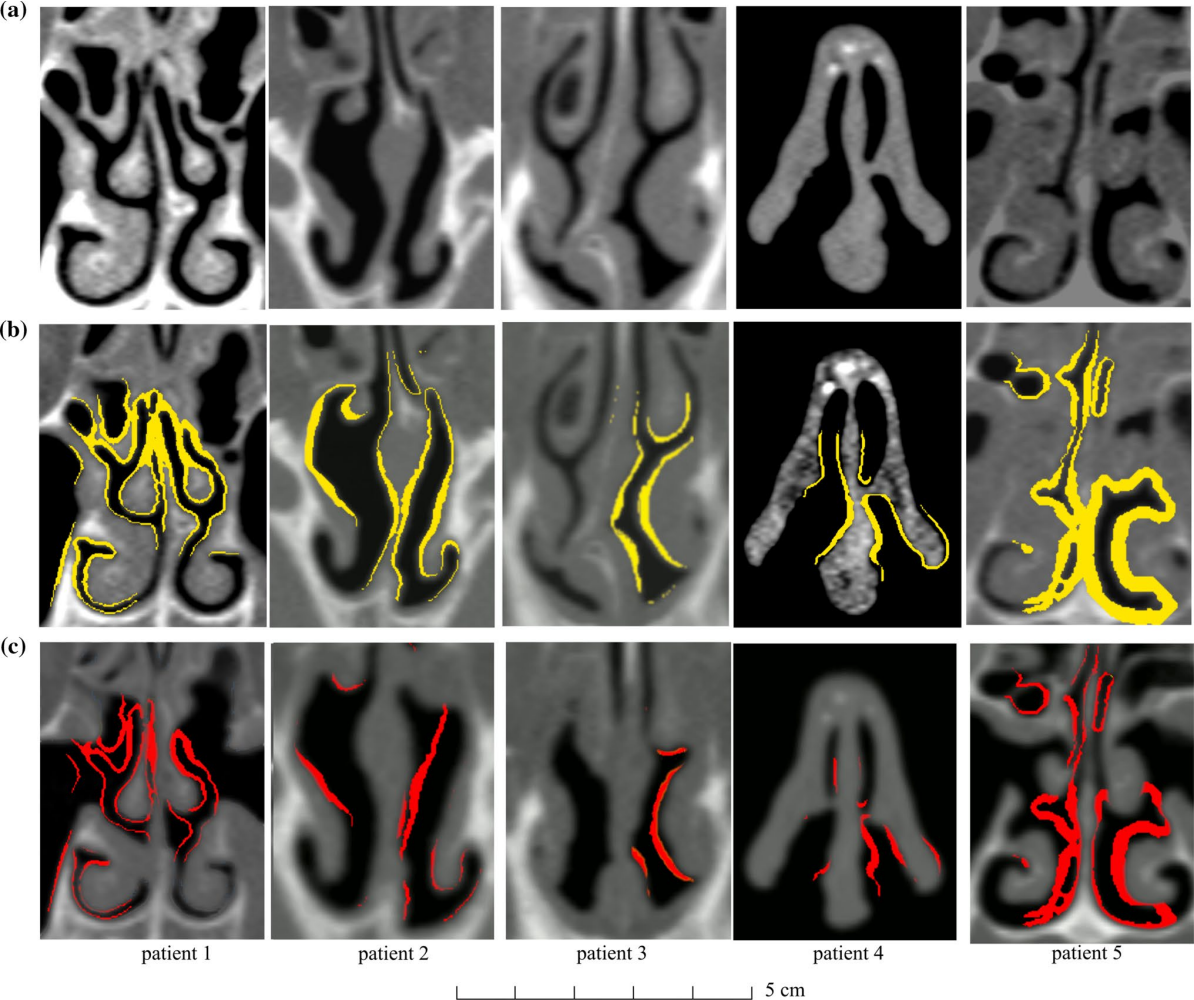
Coronal scans of nasal cavities. **a** Pre-surgery CT data. **b** Pre-surgery CT data and calculated resection areas in yellow. **c** Subtraction of registered post-surgery and predicted resection CT data, i.e. actually resected volumes (*red*)

Postoperative CT data were LB simulated for cross section comparison, Fig. 6. The overall pressure drop $\Delta p$ between nostrils and nasopharynx on pre-, post-, and virtual surgery data sets are summarized in Table 2. The change predicted by the surgical planning method (*p*_pre_ – *p*_virtual surgery_) is related to results based on the pre-, and postoperative LB simulations. The ratio

1 $$ \Delta \Pi : = \frac{{p_{{{\text{pre}}\;{\text{surgery}}}} - p_{{{\text{virtual}}\;{\text{surgery}}}} }}{{p_{{{\text{pre }}\;{\text{surgery}}}} - p_{{{\text{post}}\;{\text{surgery}}}} }} $$

relates planned to achieved results.

**Table 2:** Simulated pressure drop in Pa between nostrils and nasopharynx of the nasal cavity with total flow rate of 600 ml/s through both nostrils

	Patient 1 $\Delta p$ [Pa]	Patient 2 $\Delta p$ [Pa]	Patient 3 $\Delta p$ [Pa]	Patient 4 $\Delta p$ [Pa]	Patient 5 $\Delta p$ [Pa]
Pre	−67	−41	−69	−40	−163
Virtual	−27	−22	−30	−25	−34
Post	−35	−30	−90	−21	−36
$\Delta\Pi$	1.25	1.72	1.85	0.78	1.02

*Pre* pre-operatively; *post* postoperatively; *virtual* simulated. $\Delta\Pi$ is defined in the text

Virtual surgery LB solutions with a local critical pressure gradient of −1.1 Pa/mm yielded an average pressure drop between nostrils and nasopharynx of −27.6 Pa. Pre-surgery LB simulations of patients 2 and 4 showed overall pressure drops of ~ 40 Pa between nostril and nasopharynx, compared −36 Pa, the average of five healthy individuals. Most of the HPGRs were found at the vestibulum nasi; at patient 2 only a small amount of HPGRs was determined at the turbinate. According to the pre-surgery pressure drop in Table 2 and Fig. 5, patients 1 and 3 seem to have a “medium-severe breathing problem”; patient 5 the “most severe” problem with a too small airflow cross section throughout the nasal channel.

### Optimization results

**Fig. 7:**
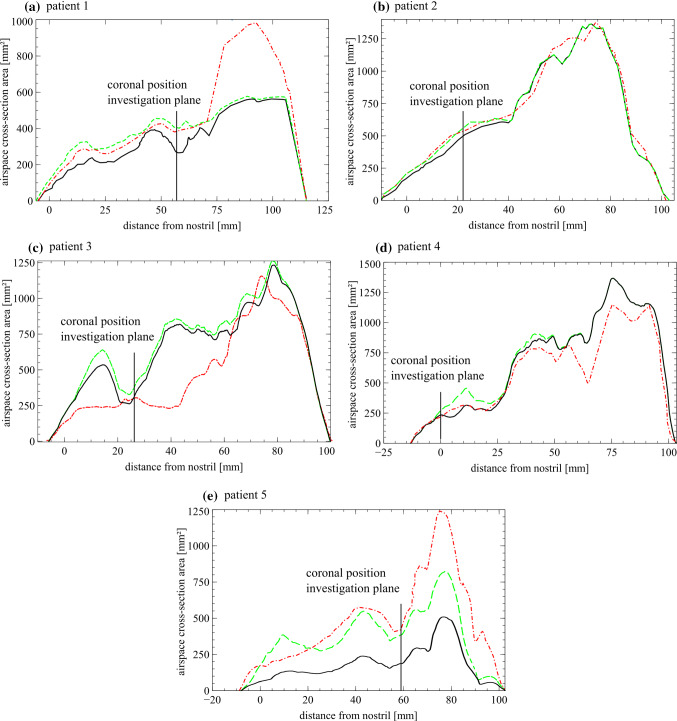
Airspace cross-sectional area versus coronal distance from the nostrils. Panels **a**–**e** show results of patient 1–5.  pre-surgery,  virtual surgery and  post-surgery

**Fig. 10:**
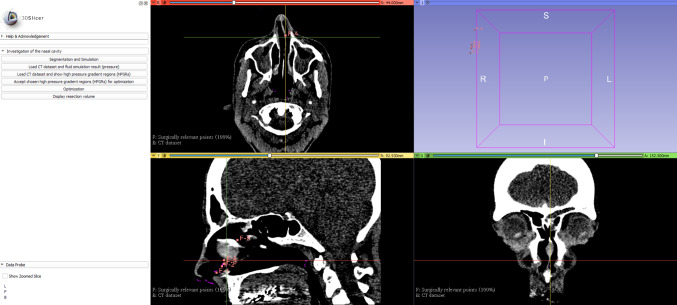
CT data and high pressure gradient regions (HPGRs, *magenta*). HPGRs for optimization are selected with the 3D-Slicer fiducial localization tool. Button naming is self-explanatory

Figure 6 shows the coronal slices defined in Fig. 5. Background images are either based on pre- (a) or postoperative (c) CT data. (b) shows the calculated resection volume, “virtual surgery” in yellow. Between 10–29 optimization iterations were necessary to reach the optimum. The actually resected volume is the Boolean difference between initial pre-surgery CT data and the final optimization result. Every optimization step takes about 3 min of computational time. Red surfaces show predicted resection areas that were actually resected. Patient 1 had a nasal airflow problem at the middle meatus, the ostium and the duct of the maxillary sinus. Optimization suggested resection there. The postoperative CT shows an increased airflow cross section. In patient 2 the nasal septum was straightened. Similar airflow cross section between virtual surgery and post-surgery was determined (see Table 3). In patient 3 optimization suggests an air-flow cross-section increase on the left nasal cavity, which was also confirmed by postoperative CT dataset. The right nasal cavity was not changed by optimization; however, in the postoperative CT dataset airflow cross section is increased there, too. Furthermore, postoperative CT data of patient 3 show a swollen middle nasal concha. The cross section was similar to the pre-surgery CT dataset. For patient 4 only minor corrections are performed close to the nostrils. Patient 5 had a nasal airflow problem at the inferior nasal meatus, which is also confirmed by the postoperative CT. Pre-surgery CT data revealed a reduced airflow cross section due to a reactively swollen nasal mucosa, a short-term clinical side effect. Figure 7 shows the airspace cross-sectional area versus coronal distance from the nostrils. For the cross-sectional area evaluation, the sphere at the nostrils, the outlet cuboid, the frontal sinus and the maxillary sinuses were not considered. The coronal investigation planes (Figs. 5, 6) of patients 1 to 5 are at coronal distances from the nostril of 57, 22, 26, 0 and 59 mm, respectively. In the post-surgery model at patient 3, at coronal distance 25–65 mm from nostril, the swollen state of the nasal cavity is identifiable due to a reduced airspace cross-sectional area. Results at coronal distance > 75 mm show differences between pre–post airspace cross-sectional areas partly due to the simple segmentation approach implemented. Here the sphenoid sinus was not removed from the investigation as no coronal investigation plane was positioned there; it was removed surgically.

**Table 3:** Air flow cross section at selected investigation planes (Fig. 5)

	Patient 1 *A* [mm^2^]	Patient 2 *A* [mm^2^]	Patient 3 *A* [mm^2^]	Patient 4 *A* [mm^2^]	Patient 5 *A* [mm^2^]
Pre	258	471	287	236	170
Virtual	412	532	320	262	381
Post	370	522	295	228	408
$\Delta\Pi$	1.11	1.02	1.08	1.15	0.93

Coronal slice positions: Patient 1 and 5: the posterior part of the nasal cavity, including the tails of the turbinates; Patient 2 and 3: anterior part of the nasal cavity including the heads of the turbinates and the infundibulum; Patient 4: area underneath the bony and cartilaginous vault, the attic; [24]

Table 3 shows the airspace cross-sectional area (A) evaluations on the investigation planes of Fig. 6. Simulated resection volume agrees within 15% with postoperative data.

Coronal slice positions: Patient 1 and 5: the posterior part of the nasal cavity, including the tails of the turbinates; Patient 2 and 3: anterior part of the nasal cavity including the heads of the turbinates and the infundibulum; Patient 4: area underneath the bony and cartilaginous vault, the attic; [24]

## Discussion

The findings show that CFD might have potential for planning of surgery to improve nasal breathing. Extending rhinomanometry, acoustic rhinometry and rhinoscopy [25], CFD could be an additional tool to determine the resection volume preoperatively.

The nasal passages have a multitude of functions [25]; there is evidence that mucosal cooling could contribute to the subjective perception of nasal airway obstruction [26–28]. The complex interaction between mucosal cooling and the surgical treatment of nasal airways is not fully understood and could be eventually responsible for the relatively unsatisfactory subjective outcome for this type of surgery [3, 4]. This work builds on two studies: one to find a surgery criterion on base of HPGRs for optimization, and two, the determination of a sufficient numbers of streamlines. The first focuses on the shape of the nasal airflow passage and the fluid flow boundary conditions. On five CT data sets from healthy persons a pressure drop criterion was developed. Averaging the findings of flow simulations of CT data of normal anatomy showed that a critical pressure gradient with −1.1 Pa/mm at flow rates of 600 ml/s through both nostrils is apt (Fig. 3). With a minimum of 25 HPGRs a wide range of pressure differences can be covered, see Fig. 3. Findings of diseased patients are all found left and above the selected criterion, healthy patients are all localized above and to the right of the criterion, see Fig. 3. This is somehow arbitrary, but it is deemed an useful criterion in the absence of others. Secondly, determining the best suited number of streamlines to detect the resection volume for each iteration during optimization. 9000 streamlines yield a stationary estimated resection volume, see Fig. 4. Manual selection of HPGRs is not possible with our computing environment. HPGRs calculation for 9000 streamlines needs 5 min, one LB simulation only 3 min. Computing 532 streamlines covers > 54% of the overall resection volume within one optimization iteration in less than 20 s. The positions of calculated HPGRs are in good accordance with clinically identified intranasal locations [29].

During optimization OC was chosen a cube of 10 × 10 × 10 mm^3^ arbitrarily but in adequate relation to the anatomy studied and to intersect the nasal airway passage and anatomy. The numbers of streamlines and the dimension of OC can be varied—at the expense of computation time and usability but showed promising results in this study.

Optimization was terminated when < 5 HPGRs, at least a reduction of HPGRs to 20% was reached. Termination at 0 HPGRs was not successful as here the whole intranasal airway and segmented anatomy had to be optimized. Virtual surgery tools to correct the nasal septum are available [14, 15] that build on non-pathologic CT data, however without considering critical pressure gradients and iterative optimization to increase the airflow cross section around points of surgical relevance. In contrast to [14], the presented approach is not limited to the nasal septum. At the current stage, the presented solution has certain restrictions regarding

- the final shape of nasal airway passage to achieve,
- which surgical approaches are being used,
- the handling of anatomy to assure a medically correct “surgery”,
- the absence of collision criteria of predicted resection volumes to avoid “complications” like a perforated septum (e.g. Fig. 6, Patient 5).
- the ease of use and anatomically correct segmentation of the nasal airway especially between nasal fossae and the sinuses.
- CFD simulations, where D3Q19 is known to violate rotational invariance [30, 31] and might cause numerical errors. A validation experiment, however, showed acceptable agreements between simulation and LDA measurement data [12] so that the use of D3Q19 seems justifiable.

Results show similarities between CFD optimizations and postoperative CT datasets (Fig. 7, Tables 2, 3).

Overall, the optimization process took less than 1.5 h for each of the five patients with the following steps:

- Import CT data
- Nasal airflow segmentation and simulation
- Streamline computation, determination of initial HPGRs
- Manual selection of HPGRs
- Optimization of nasal airflow
- Display resection volume

The used flow rate of 600 ml/s is slightly higher than breathing at rest or light physical activity (500 ml/s) [32]. Earlier [12], a comparison of laser Doppler anemometry measurements and CFD simulations of nasal airflow with maximum rhinomanometry flow rate of 1600 ml/s was performed. The flow rate had to be reduced in Sailfish CFD to reach stable simulations down to 600 ml/s. LES turbulence model was used to simulate the transitional features of nasal airflow [20]; the results were validated by LDA measurements and a mesh convergence study [12]. Spatial resolution and lattice element (used: D3Q19) was limited by GPU RAM with 11 GB. At a flow rate of 600 ml/s for both nostrils LB fluid flow simulations were numerically stable in all optimization iterations. In order to reach a full-fledged patient simulation one would have to include different airflows (to model physical activities) and physiologic and mechanical (swelling) tissue conditions. This would be out of the scope of this investigation. Moreover, the influence of the nasal cycle on the airflow cross section during imaging was not controllable in the presented study.

Furthermore, we did not consider surgeon knowledge and anatomy. While the simulations presented in this article could be a step toward the development of a clinical virtual surgery planning tool, it does not account for all the complexities of nasal surgery [33, 34]. For example, Fig. 6 shows that the algorithm recommended trimming the lateral wall of the right cavity of patient 2 even though the cavity was already wide at that location.

Sailfish LB simulations were generally numerically stable [6], divergence occurred when HPGRs and optimization region were close to inlet/outlet.

Further work should include improving the optimizer to restrict resection to anatomically feasible and surgically accessible points and to eventually allow computational straightening of deformed septa. The user interface was developed with surgeon feedback and is shown in Appendix A. A future clinical trial is required to fully assess the clinical validity of the methods developed here.

## Figures and Tables

**Fig. 8:**
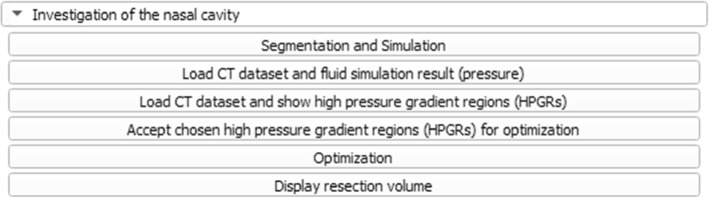
Command buttons of the graphical user interface; button naming is self-explanatory

**Fig. 9:**
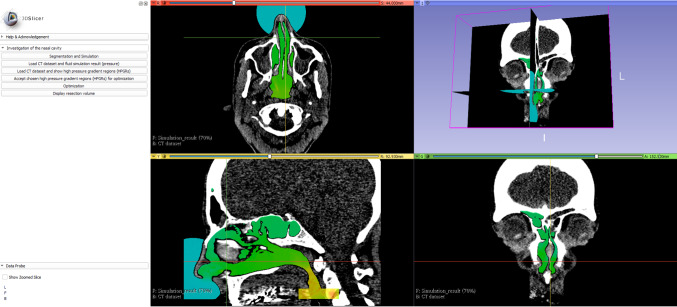
CT data and CFD simulation results for pressure shown in color without scale

**Fig. 11:**
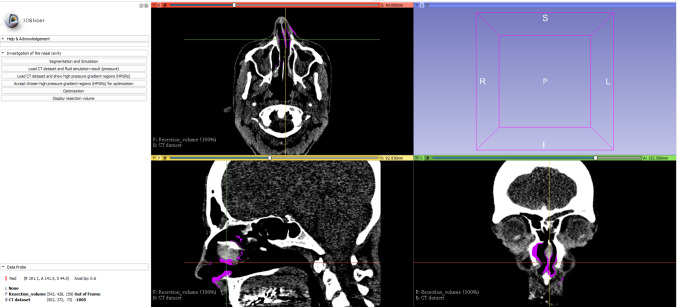
CT data and calculated resection volume (*violet*)

## Appendix A: graphical user interfaces

See Figs. 8, 9, 10, and 11.
